# Clinical utility of genetic tests for inherited hypertrophic and dilated cardiomyopathies

**DOI:** 10.1186/1476-7120-6-62

**Published:** 2008-12-19

**Authors:** Maria Giovanna Colombo, Nicoletta Botto, Simona Vittorini, Umberto Paradossi, Maria Grazia Andreassi

**Affiliations:** 1 Genetics Research Unit G. Monasterio Foundation, CNR-Regione Toscana, CNR Institute of Clinical Physiology, Massa, Italy

## Abstract

Genetic testing has become an increasingly important part of medical practice for heritable form of cardiomyopathies. Hypertrophic cardiomyopathy and about 50% of idiopathic dilatative cardiomyopathy are familial diseases, with an autosomal dominant pattern of inheritance.

Some genotype-phenotype correlations can provide important information to target DNA analyses in specific genes. Genetic testing may clarify diagnosis and help the optimal treatment strategies for more malignant phenotypes. In addition, genetic screening of first-degree relatives can help early identification and diagnosis of individuals at greatest risk for developing cardiomyopathy, allowing to focus clinical resources on high-risk family members.

This paper provides a concise overview of the genetic etiology as well as the clinical utilities and limitations of genetic testing for the heritable cardiomyopathies.

## Background

The success of the Human Genome Project and the recent discoveries in the area of genetics promise to significantly change the clinical practice of cardiology, providing new tools for more accurate diagnosis and prognosis of disease as well as for a better prediction about health risks for the family [1,2].

Rapid advances in the technology and reduction in the cost of DNA sequencing have led to increasingly rapid translation of genomic information into clinical applications [3]. As a consequence the number of genetic tests is growing, and becoming currently available for clinical testing [4]. However, genetic tests are generally time-consuming and expensive, and they should be used with sufficient consideration of their necessity and value in managing the patient's condition. Therefore, it is imperative that cardiologists know the basis for genetic cardiovascular disorders and the medical implications of these defects in order to improve their expertise as well as to ensure an appropriate practical use of genetic tests in the clinical setting. The purpose of this paper is to provide a concise overview of the genetic etiology as well as the clinical utilities and limitations of genetic testing for the heritable form of hypertrophic and dilated cardiomyopathies.

## The genetics of cardiomyopathies

**Figure 1:**
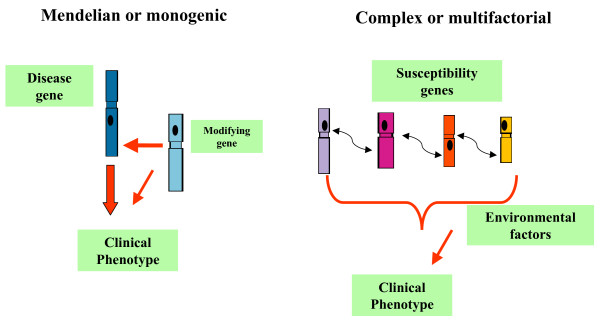
**Single gene disorders or complex traits**. A single rare mutation can fully account for a Mendelian disease; clinical variability can be, however, observed even for determined monogenic disease, and this variability may itself involve genetic factors, the so-called modifier genes. In contrast to monogenic traits, complex traits have many contributing genes and non-genetic influences.

Diseases identified as "genetic" can be typically classified into two categories: Mendelian diseases and multifactorial diseases. Mendelian diseases or monogenic diseases are rare, and only one mutation in a given gene is responsible for inheritance of the disease in a given family. Genetic diseases, in the second group, occur more commonly in the population and are often recognized as "running in families" [5]. The genetic model underlying a multifactorial disease is often complex since it may be related to the interaction or additive effect of multiple genes as well as to the presence or absence of environmental factors (Figure 1). Congenital heart disease, coronary heart disease, venous thrombosis, and diabetes mellitus fall into this category.

Heritable hypertrophic and dilated cardiomyopathies are monogenic diseases, caused by mutations in key genes that lead to the absence or abnormality of myocardial proteins [5].

Disease-causing gene mutations have been identified in approximately two-thirds of cases of hypertrophic cardiomyopathy (HCM) and about 50% of idiopathic dilated cardiomyopathy (DCM).

Various types of mutations can occur in DNA, including non-sense (stop codons), missense mutations (causing aminoacid substitution) and splice-site. Mostly, newly detected mutations for heritable cardiovascular disorders are missense. For these mutations, it is difficult to establish their pathogenicity, unless specific functional test are available. Presently, pathogenicity is presumed when the substitution affects a very conserved sequence through evolution, or it was reportedly associated to disease in independent patients.

The accurate reconstruction of family history is crucial element for diagnosis of genetic cardiomyopathy [6].

The family history should encompass at least 3 generations with a careful and complete history about the family members, including demographic and medical information [7].

**Figure 2:**
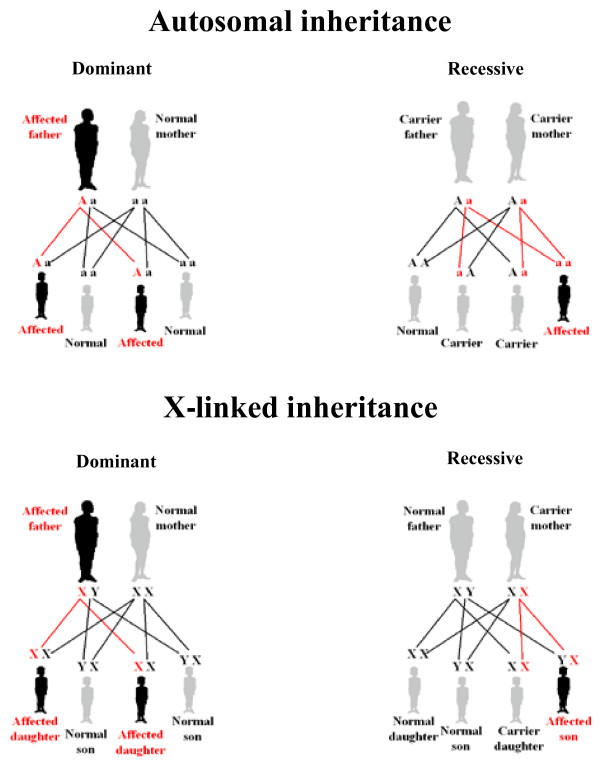
**Autosomal and X-linked patterns of inheritance**. In autosomal dominant inheritance, family history typically reveals that the disorder is usually present in every generation, and there is a 50% chance of inheriting the mutation. In autosomal recessive inheritance, the condition appears to "skip" generations. Parents of an affected have a 25% chance of having an affected child and a 50% chance of having a carrier child in each pregnancy. In X-linked dominant inheritance, all daughters of an affected man are affected, sons and daughters of carrier women have a 50% risk of being affected. In X-linked recessive inheritance, there is a 50% chance that each son of a carrier woman will also be affected. No male-male transmission is observed.

The family history may provide additional relevant information, such as age of onset and penetrance, and help to identify the patterns of inheritance (Figure 2).

In addition, information of cardiovascular tests and procedures (echocardiography, electrocardiography, Holter monitoring, implantable cardioverter-defibrillator, heart transplant) should always be recorded as part of the family history [6].

However, inherited cardiomyopathies show a wide range of clinical presentation within the same family, with incomplete and age-dependent penetrance. Genotype-phenotype relations are complex and not yet completely understood. Considerable phenotypic heterogeneity exists even among individuals who have identical mutations at the disease-causing locus.

Conversely, mutations in different genes can result in a similar phenotype. The incompleteness of genotype-phenotype correlations has limited the use of genetic testing in clinical practice. In selected cases, DNA target analysis has, however, potential clinical value, and genetic testing should be done in patients and families.

## Hypertrophic cardiomyopathy (HCM)

Hypertrophic cardiomyopathy (HCM) is a relatively common and autosomal dominant genetic heart disease (1:500 of the general population), typically diagnosed by unexplained left ventricular hypertrophy with 2-dimensional echocardiography (or alternatively with cardiac magnetic resonance imaging) [8,9]. Left ventricular hypertrophy, often disproportionately affecting the ventricular septum, can range from mild (~13–15 mm) to severe (30–60 mm). The clinical spectrum of HCM is diverse, ranging from asymptomatic individuals to those with disabling symptoms of heart failure, exercise intolerance, arrhythmias and chest pain [9].

Moreover, HCM is the most common cause of sudden cardiac death (SCD) in the young (including trained athletes), who are often unaware of their underlying condition. Early diagnosis of HCM is important, since at-risk individuals may be advised not to participate in competitive sports and should undergo regular cardiac screening to assess the risk of sudden cardiac death [10].

HCM is caused by a variety of mutations in genes encoding contractile proteins of the cardiac sarcomere, especially in cardiac myosin heavy chain beta (*MYH7*), myosin binding protein C (*MYBPC3*), and cardiac troponin T (*TNNT2*) [8-11]. To date, over 700 individual mutations have been identified (for a list of gene mutations see .

Mutations in the genes coding for 3 sarcomeric proteins (*MYH7*; *MYBPC3*; *TNNT2*), most commonly are estimated to account for about 60% of all familial cases of HCM (Table 1). Clinical testing for variants in most of these genes is available and can provide valuable therapeutic and prognostic information.

**Table 1:** Gene mutations in sarcomere-related genes in HCM.

**Gene**	**Protein**	**Frequency (%)**
*MYH7*	Cardiac myosin heavy chain beta	**30–50**

*MYBPC3*	Myosin binding protein C	**20–40**

*TNNT2*	Cardiac troponin T	**5–20**

*TNNI3*	Cardiac troponin I	**<5**

*TPM1*	α-tropomyosin	**<5**

*MYL2*	Cardiac myosin light chain 2, regulatory	**<3**

*MYL3*	Cardiac myosin light chain 3, essential	**<1**

*ACTC*	Cardiac actin	**<1**

*TTN*	Titin	**rare**

*MYH6*	α-myosin heavy chain	**rare**

Overall, clinical diversity of HCM can reflect the broad spectrum of underlying molecular cause.

Some specific mutations have been recognized to be associated with strong clinical effect [10-15]. In particular, some missense mutations in the gene of the *MYH7 *(R403Q, R453C, G716R and R719W) were associated with early onset and poor clinical prognosis, including sudden death. Moreover, mutations in the *TNNT2 *gene (R92Q and ΔE160) have been associated with high incidence of cardiac sudden death in young men, even with mild hypertrophy. In contrast, patients with mutations in the *MYBPC3 *can have a late onset and a relatively good prognosis [8-11].

Nevertheless, defining precise genotype-phenotype correlations has been limited by the low frequency of each mutation. Studies of HCM families have also shown the presence of clinical variability among individual with identical mutations. Moreover, the clinical presentation within a given kindred may vary between family members, with mild clinical symptoms manifested in one relative and early-onset heart disease in another. Such interfamilial variable expressivity may be explained by genetic and environmental modifying factors. The identification of other genetic alterations that might play a role in modulating the presentation of disease is, thus, crucial in order to improve gene-based diagnosis.

Recently, nonsarcomeric protein mutations in 2 genes (gamma-2 regulatory subunit of AMP-activated protein kinase, *PRKAG2*, and lysosome-associated membrane protein 2, *LAMP2*) involved in glycogen accumulation in cardiac myocytes have also been reported to be responsible for a less common type of HCM, known as metabolic HCM [13]. Clinical features of metabolic HCM are also the high prevalence of electrical abnormalities (ventricular pre-excitation and atrial fibrillation) and faster progression from hypertrophy to dilation and severe heart failure with respect to HCM. Mutations in the *PRKAG2 *or in *LAMP2 *genes are believed to account for about 1% of all HCM and for up to 50% of HCM with ventricular preexcitation [16-18].

**Figure 3:**
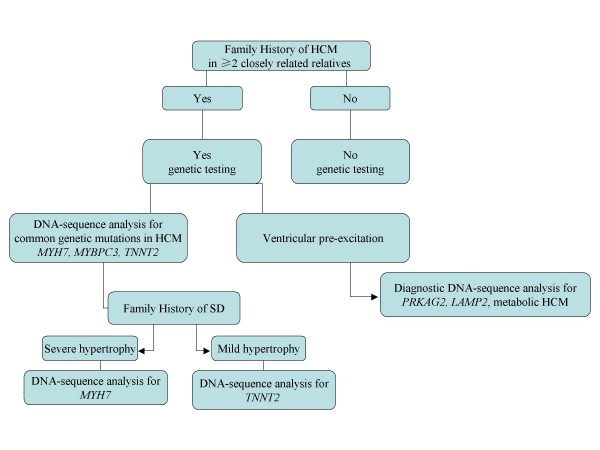
**Potential guidelines for genetic test selection in inherited hypertrophic cardiomyopathy**.

Genetic testing for mutant genes is the most definitive method for establishing the diagnosis of HCM, and some genotype-phenotype correlations can be useful to address DNA analyses in specific genes. For example, HCM with late onset, good prognosis and mild hypertrophy can help to target DNA analysis for *MYBPC3 *mutations. Conversely, the presence a more malignant phenotype with a high risk of SCD, may guide genetic screening for *MYH7 *mutations in the presence of severe hypertrophy or *TNNT2 *mutations if the degree of hypertrophy is mild (Figure 3).

However, caution is required because genotype-phenotype correlations are based on a small population. In addition, the coinheritance of compound mutations (more than 1 mutation in a single gene, or mutations in 2 different genes) is more common than might be expected and can explain why different families that appear to have the same mutation can behave differently.

## Dilated cardiomyopathy (DCM)

Dilated cardiomyopathy (DCM) is a common (estimated 1:2500 persons) and largely irreversible form of heart muscle disease; it is the third most common cause of heart failure in the young and a major cause of heart transplantation [9].

DCM, diagnosed by 2-dimensional echocardiography, is characterized by left ventricular dilation and impaired left ventricular systolic function, often with involvement of the right ventricle, which then lead to ventricular and supraventricular arrhythmias, conduction system abnormalities, thromboembolism, and sudden or heart failure-related death.

DCM may derive from a particularly broad range of primary causes, including infectious agents, toxins, chronic excessive consumption of alcohol, chemotherapeutic agents, autoimmune, neuromuscular disorders, mitochondrial, metabolic and endocrine disorders.

Approximately one half of patients with the disease are found to have "idiopathic" DCM, indicating that the cause for the condition cannot be determined. About one-third to one-half of "idiopathic" DCM cases has a positive family history in ≥ 2 closely related relatives. These patients are considered to have familial dilated cardiomyopathy [19].

It is noteworthy that familial DCM is clinically and diagnostically the same as other forms of DCM, so careful attention to family history is essential.

Inheritance patterns of familial DCM include autosomal dominant, autosomal recessive and X-linked. The autosomal dominant forms are the most common inheritance accounting for about 85–90% of cases.

DCM exhibits high genetic heterogeneity as mutations in > 20 genes have been associated with the disease, such as desmin, tafazzin, *δ*-sarcoglycan, dystrophin, and metavinculin, and nuclear envelope proteins such as emerin and lamin A/C [20].

Mutations in the sarcomere genes, which are responsible for causing HCM, are also associated with DCM. To date, sarcomere gene mutations (*MYBPC3; MYH7;TNNT2; *cardiac troponin I, *TNNI3; α-tropomyosin,TPM1; *cardiac actin, *ACTC*) account for approximately 10–16% of familial DCM [21,22].

At the present time, genetic screening in all known disease genes is not possible. However, lamin A/C (*LMNA*) is the most frequent disease associated gene for familial DCM with conduction system disease.

**Figure 4:**
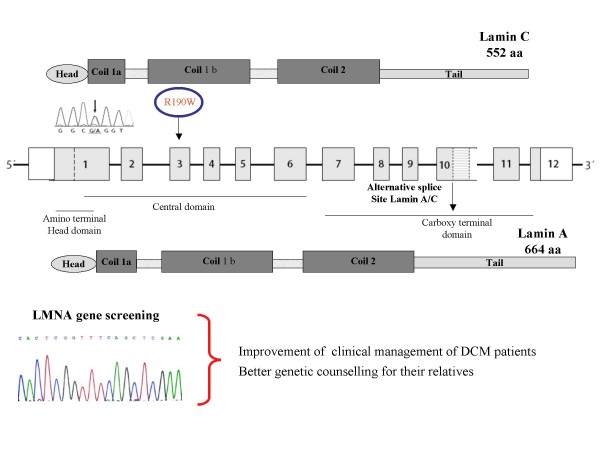
**Schematic representation of the genomic organization of the *LMNA *gene and localization of most prevalent *LMNA *mutation hot spots**. Exon 11 and part of exon 12 encode for lamin A (664 aminoacids) while the alternatively spliced part of exon 10 encodes for the lamin C (572 aminoacids) isoform.

The *LMNA *gene encodes the two differentially spliced proteins lamin A and lamin C, the major components of the nuclear lamina, which localizes at the nucleoplasmic surface of the inner nuclear membrane as a meshwork structure (Figure 4) [23,24]. Lamin interacts directly with the chromatin and also with the integral proteins of the inner nuclear membrane, thereby playing a role in maintaining the structural integrity and spatial organization of other inner nuclear membrane. During the last years, clinically distinct disease phenotypes have been attributed to *LMNA *mutations-termed "laminopathies"-ranging from accelerated aging disorders to striated muscle diseases like muscular dystrophy and cardiomyopathy [24,25].

**Figure 5:**
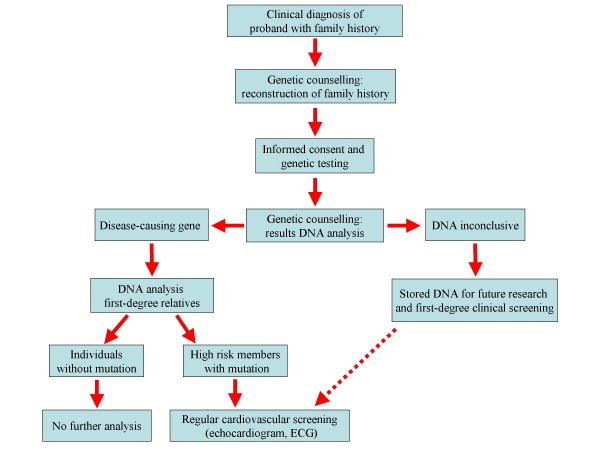
**Flow chart showing the clinical management and the genetic counselling for the heritable cardiomyopathies by a specialised cardiogenetic team**.

Indeed, the *LMNA *gene is involved in up to 30–50% of patients with cardiac conduction disorders and DCM [25-32]. Although mutations causing DCM can occur almost anywhere in *LMNA*, the domain coil 1B seems to be most frequently affected (Figure 5).

The most prevalent *LMNA *mutation hot spot in familial DCM in Europe is codon 190 [32], a missense mutation (R190W) initially described by Arbustini and colleagues in an Italian family with severe DCM and SCD [30]. Subsequently, this variant was identified in DCM patients from other European countries, and even from Korea [33-35].

A complete list for *LMNA *mutations causing DCM is available in: the Human Gene Mutation Database , the locus specific *LMNA *database , and the Leiden Muscular Dystrophy pages .

A meta-analysis of clinical characteristics of *LMNA *mutation carriers revealed that *LMNA *mutations carry a high risk of sudden death, and that this risk does not differ between subjects with predominantly cardiac or neuromuscular disease [36].

Pasotti et al. have recently shown that dilated cardiomyopathies caused by *LMNA *gene defects are highly penetrant, adult onset, malignant diseases characterized by a high rate of heart failure and life-threatening arrhythmias, predicted by New York Heart Association functional class, competitive sport activity, and type of mutation [37].

All these studies indicate that DCM patients with *LMNA *mutations show remarkable homogenous clinical phenotypes with sinoatrial and atrioventricular node dysfunction, heart block commonly requiring pacemakers, atrial fibrillation, other supraventricular arrhythmias, sudden cardiac death, and a malignant course with heart failure necessitating heart transplantation.

Therefore, it is highly recommended to screen *LMNA *for genetic variants when such clinical features are present in a DCM patient.

The *LMNA *genetic testing may allow useful diagnosis of mutations clearly correlated with a worse prognosis as well as identify early "presymptomatic" relatives at greatest risk for developing DCM (Figure 5).

## Potential benefits, disadvantages and appropriateness of genetic testing

Genetic testing is often the best way to confirm a diagnosis in a patient with HCM and DCM, as well as to provide risk estimates for asymptomatic patients. However, genetic tests remain generally expensive technologies that are labour-intensive and time-consuming.

Rapid advances in the technology and reduction in the cost of DNA sequencing are expected to decrease the costs and, thus, increase the use of genetic testing, perhaps even within the next years. At the present time, costs may vary considerably (from several hundreds to thousands Euro €) depending on the number of genes and nucleotides examined. For testing gene in the first family member, sequencing gene now cost on the order of €1500–€4000. If a mutation is identified, other family members may be offered confirmatory testing at a reduced rate that is around €250.

Therefore, routine and extensive genetic screening is impractical because of the genetic heterogeneity of cardiomyopathies. Genetic testing is not appropriate for every patient, but it should be used in selected cases, such as patients with an established family history of severely affected relatives and at high risk of worse prognosis.

For example, clinical DNA testing for gene mutations known to be associated with a more malignant phenotype (e.g. *TNNT2 *in HCM and *LMNA *in DCM) can confirm the diagnosis and help the cardiologist to stratify the risk of patient.

However, the utility of DNA diagnosis for risk stratification is expected to be limited by the genetic and allelic heterogeneity of cardiomyopathies. A single gene mutation does not by itself fully explain the development of the clinical phenotype. For example, evidence is accumulating that the combined effect of more than one disease-associated mutation or genetic polymorphisms, which contribute to cardiovascular performance, may affect penetrance and severity of the disease in many families.

Anyway, it should be emphasized that genetic screening is superior to clinical with respect to specificity of identification of family members at high risk [38,39].

Genetic testing unambiguously allows early identification and diagnosis of individuals at greatest risk for developing cardiomyopathy, allowing to focus clinical resources on high-risk family members.

In addition, it is extremely important that family members receive careful counselling both before and after testing on the potential risks. Relative may carry the mutation but be asymptomatic and the mutation may merely be a predisposing factor to disease in the presence of other factors, and so its presence alone does not allow accurate prediction of phenotype or prognosis. However, if a mutation is identified in asymptomatic individual, regular clinical cardiovascular screening (echocardiogram, ECG) is recommended to detect the first signs of disease that may be diminished by early treatment.

If family members are found not to carry that mutation, they can be definitively diagnosed as unaffected, and the need for serial follow up becomes unnecessary. In this case, they can be reassured that neither they nor their offspring will be at higher risk compared to the general population to develop these disorders.

A specialised cardiogenetic team consisting of clinical geneticists and cardiologists should work together in order to provide the most relevant information to the patients and the relatives, as summarized in Figure 5.

For instance, patients with suspected inherited cardiomyopathy are referred to the cardiogenetic team to ascertain the family history and discuss the importance of molecular analysis. After informed consent, blood samples are drawn for DNA analysis. Subsequently, further consultation with the geneticists can help clarify the interpretation of the results of the DNA analysis. If the disease-causing gene cannot be predicted or investigated, DNA is stored for future research and screening, if permitted by the patient. If a pathogenic mutation is detected in the proband, the team provide genetic counselling for family members with subsequent DNA testing when family members decide to undergo genetic screening.

## Conclusion

Genetic testing has become an increasingly important part of medical practice for heritable form of cardiomyopathies. HCM and about 50% of idiopathic DCM are familial diseases. They generally show an autosomal dominant pattern of inheritance and have underlying mutations in specific genes; some of these mutations are known to be associated with a more malignant phenotype. Some genotype-phenotype correlations can provide important information to target DNA analyses.

Therefore, it is important that physicians develop a specific knowledge for an appropriate practical use of genetic screening. In every case, it will crucial to establish a direct collaboration between geneticists and cardiologists in a combined clinical setting (cardiogenetics team).

## Competing interests

The authors declare that they have no competing interests.

## Authors' contributions

All authors have been involved in writing the manuscript and have approved it in its final submitted form.

## Glossary of Genetics Terminology

**- Chromosomes**: single long DNA molecule, whose regions producing a functional RNA, constitutes a gene. Somatic cells contain 46 chromosomes, existing as 23 different pairs. One of the chromosomes in each pair is inherited from the mother, while the other is inherited from the father.

**- Gene**: complex functional DNA unit on the chromosome that provides instructions for building a protein.

**- Allele**: a form of a gene. Because chromosomes are paired, subjects usually carry 2 alleles of each gene.

**- Homozygote**: an individual with 2 identical alleles of a specific gene.

**- Heterozygote**: an individual with 2 different alleles of a specific gene.

**- Phenotype**: the observed characteristics of an individual as defined by genotype.

**- Penetrance**: the percentage of individuals with a disease-genotype who express the associated phenotype. Complete penetrance occurs when all individuals who carry the disease-genotype express the associated phenotype.

**- Variable expressivity**: the range of different phenotypes (from mild to severe) associated with a particular genotype. Family members may share the same genotype but present different symptoms or severity.

## References

[B1] Collins FS, McKusick VA (2001). Implications of the human genome project for medical science. JAMA.

[B2] McPherson E (2006). Genetic diagnosis and testing in clinical practice. Clin Med Res.

[B3] Collins FS, Green ED, Guttmacher AE, Guyer MS (2003). A Vision for the Future of Genomics Research. Nature.

[B4] Cowan L, Morales A, Dagua J, Hershberger R (2008). Genetic Testing and Genetic Counseling in Cardiovascular Genetic Medicine: Overview and Preliminary Recommendations. Congest Heart Fail.

[B5] Robin NH, Tabereaux PB, Benza R, Korf BR (2007). Genetic testing in cardiovascular disease. J Am Coll Cardiol.

[B6] Morales A, Cowan J, Dagua J, Hershberger RE (2008). Family history: an essential tool for cardiovascular genetic medicine. Congest Heart Fail.

[B7] Wattendorf DJ, Hadley DW (2005). Family history: the three-generation pedigree. Am Fam Physician.

[B8] Maron BJ, McKenna WJ, Danielson GK, Kappenberger LJ, Kuhn HJ, Seidman CE, Shah PM, Spencer WH, Spirito P, Ten Cate FJ, Wigle ED, Task Force on Clinical Expert Consensus Documents. American College of Cardiology; Committee for Practice Guidelines. European Society of Cardiology. Task Force on Clinical Expert Consensus Documents. American College of Cardiology; Committee for Practice Guidelines; European Society of Cardiology (2003). American College of Cardiology/European Society of Cardiology: Clinical expert consensus document on hypertrophic cardiomyopathy. A report of the American College of Cardiology Foundation Task Force on Clinical Expert Consensus Documents and the European Society of Cardiology Committee for Practice Guidelines. J Am Coll Cardiol.

[B9] Maron BJ, Towbin JA, Thiene G, Antzelevitch C, Corrado D, Arnett D, Moss AJ, Seidman CE, Young JB, American Heart Association; Council on Clinical Cardiology, Heart Failure and Transplantation Committee; Quality of Care and Outcomes Research and Functional Genomics and Translational Biology Interdisciplinary Working Groups; Council on Epidemiology and Prevention (2006). Contemporary definitions and classification of the cardiomyopathies: an American Heart Association Scientific Statement from the Council on Clinical Cardiology, Heart Failure and Transplantation Committee; Quality of Care and Outcomes Research and Functional Genomics and Translational Biology Interdisciplinary Working Groups; and Council on Epidemiology and Prevention. Circulation.

[B10] Taylor MR, Carniel E, Mestroni L (2004). Familial hypertrophic cardiomyopathy: clinical features, molecular genetics and molecular genetic testing. Exp Rev Mol Diagn.

[B11] Ho CY, Seidman CE (2006). A contemporary approach to hypertrophic cardiomyopathy. Circulation.

[B12] Bos MJ, Ommen SR, Ackerman MJ (2007). Genetics of hypertrophic cardiomyopathy: one, two, or more diseases?. Curr Opin Cardiol.

[B13] Richard P, Charron P, Carrier L, Ledeuil C, Cheav T, Pichereau C, Benaiche A, Isnard R, Dubourg O, Burban M, Gueffet JP, Millaire A, Desnos M, Schwartz K, Hainque B, Komajda M, EUROGENE Heart Failure Project (2003). Hypertrophic cardiomyopathy: distribution of disease genes, spectrum of mutations, and implications for a molecular diagnosis strategy. Circulation.

[B14] Maron BJ, Olivotto I, Spirito P, Casey SA, Bellone P, Gohman TE, Graham KJ, Burton DA, Cecchi F (2000). Epidemiology of hypertrophic cardiomyopathy-related death: revisited in a large non-referral-based patient population. Circulation.

[B15] Seidman JC, Seidman C (2001). The genetic basis for cardiomyopathy: from mutation identification to mechanistic paradigms. Cell.

[B16] Maron BJ, Seidman JG, Seidman CE (2004). Proposal for contemporary screening strategies in families with hypertrophic cardiomyopathy. J Am Coll Cardiol.

[B17] Arad M, Maron BJ, Gorham JM, Johnson WH, Saul JP, Perez-Atayde AR, Spirito P, Wright GB, Kanter RJ, Seidman CE, Seidman JG (2005). Glycogen storage diseases presenting as hypertrophic cardiomyopathy. N Engl J Med.

[B18] Fanin M, Nascimbeni AC, Fulizio L, Spinazzi M, Melacini P, Angelini C (2006). Generalized lysosome-associated membrane protein-2 defect explains multisystem clinical involvement and allows leukocyte diagnostic screening in Danon disease. Am J Pathol.

[B19] Burkett EL, Hershberger RE (2005). Clinical and genetic issues in familial dilated cardiomyopathy. J Am Coll Cardiol.

[B20] Murphy RT, Starling RC (2005). Genetics and cardiomyopathy: Where are we now?. Cleve Clin J Med.

[B21] Kamisago M, Sharma SD, DePalma SR, Solomon S, Sharma P, McDonough B, Smoot L, Mullen MP, Woolf PK, Wigle ED, Seidman JG, Seidman CE (2000). Mutations in sarcomere protein genes as a cause of dilated cardiomyopathy. N Engl J Med.

[B22] Zeller R, Ivandic BT, Ehlermann P, Mucke O, Zugck C, Remppis A, Giannitsis E, Katus HA, Weichenhan D (2006). Large-scale mutation screening in patients with dilated or hypertrophic cardiomyopathy: a pilot study using DGGE. J Mol Med.

[B23] Capell BC, Collins FS (2006). Human laminopathies: nuclei gone genetically awry. Nat Rev Genet.

[B24] Worman HJ, Bonne G (2007). Laminopathies: a wide spectrum of human disease. Exp Cell Res.

[B25] Fatkin D, MacRae C, Sasaki T, Wolff MR, Porcu M, Frenneaux M, Atherton J, Vidaillet HJ, Spudich S, De Girolami U, Seidman JG, Seidman C, Muntoni F, Müehle G, Johnson W, McDonough B (1999). Missense mutations in the rod domain of the lamin A/C gene as causes of dilated cardiomyopathy and conduction-system disease. N Engl J Med.

[B26] Brodsky G, Muntoni F, Miocic S, Sinagra G, Sewry C, Mestroni L (2000). Lamin A/C gene mutation associated with dilated cardiomyopathy with variable skeletal muscle involvement. Circulation.

[B27] Becane HM, Bonne G, Varnous S, Muchir A, Ortega V, Hammouda EH, Urtizberea JA, Lavergne T, Fardeau M, Eymard B, Weber S, Schwartz K, Duboc D (2000). High incidence of sudden death with conduction system and myocardial disease due to lamins A and C gene mutation. Pacing Clin Electrophysiol.

[B28] Jakobs PM, Hanson E, Crispell KA, Toy W, Keegan H, Schilling K, Icenogle TB, Litt M, Hershberger RE (2001). Novel lamin A/C mutations in two families with dilated cardiomyopathy and conduction system disease. J Card Fail.

[B29] Hershberger RE, Hanson E, Jakobs PM, Keegan H, Coates K, Bousman S, Litt M (2002). A novel lamin A/C mutation in a family with dilated cardiomyopathy, prominent conduction system disease, and need for permanent pacemaker implantation. Am Heart J.

[B30] Arbustini E, Pilotto A, Repetto A, Repetto A, Grasso M, Negri A, Diegoli M, Campana C, Scelsi L, Baldini E, Gavazzi A, Tavazzi L (2002). Autosomal dominant dilated cardiomyopathy with atrioventricular block: a lamin A/C defect-related diseas. J Am Coll Cardiol.

[B31] van Tintelen JP, Hofstra RM, Katerberg H, Rossenbacker T, Wiesfeld AC, du Marchie Sarvaas GJ, Wilde AA, van Langen IM, Nannenberg EA, Kooi AJ van der, Kraak M, van Gelder IC, van Veldhuisen DJ, Vos Y, Berg MP van den, Working Group on Inherited Cardiac Disorders, line 27/50, Interuniversity Cardiology Institute of The Netherlands (2007). High yield of LMNA mutations in patients with dilated cardiomyopathy and/or conduction disease referred to cardiogenetics outpatient clinics. Am Heart J.

[B32] Perrot A, Hussein S, Ruppert V, Schmidt HH, Wehnert MS, Duong NT, Posch MG, Panek A, Dietz R, Kindermann I, Böhm M, Michalewska-Wludarczyk A, Richter A, Maisch B, Pankuweit S, Ozcelik C Identification of mutational hot spots in LMNA encoding lamin A/C in patients with familial dilated cardiomyopathy. Basic Res Cardiol.

[B33] Hermida-Prieto M, Monserrat L, Castro-Beiras A, Laredo R, Soler R, Peteiro J, Rodríguez E, Bouzas B, Alvarez N, Muñiz J, Crespo-Leiro M (2004). Familial dilated cardiomyopathy and isolated left ventricular noncompaction associated with lamin A/C mutations. Am J Cardiol.

[B34] Kärkkäinen S, Reissell E, Heliö T, Kaartinen M, Tuomainen P, Toivonen L, Kuusisto J, Kupari M, Nieminen MS, Laakso M, Peuhkurinen K (2006). Novel mutations in the lamin A/C gene in heart transplant recipients with end stage dilated cardiomyopathy. Heart.

[B35] Song K, Dube MP, Lim J, Hwang I, Lee I, Kim JJ (2007). Lamin A/C mutations associated with familial and sporadic cases of dilated cardiomyopathy in Koreans. Exp Mol Med.

[B36] Van Berlo JH, de Voogt WG, Kooi AJ van der, van Tintelen JP, Bonne G, Yaou RB, Duboc D, Rossenbacker T, Heidbüchel H, de Visser M, Crijns HJ, Pinto YM (2005). Meta-analysis of clinical characteristics of 299 carriers of LMNA gene mutations: Do lamin A/C mutations portend a high risk of sudden death?. J Mol Med.

[B37] Pasotti M, Klersy C, Pilotto A, Marziliano N, Rapezzi C, Serio A, Mannarino S, Gambarin F, Favalli V, Grasso M, Agozzino M, Campana C, Gavazzi A, Febo O, Marini M, Landolina M, Mortara A, Piccolo G, Viganò M, Tavazzi L, Arbustini E (2008). Long-term outcome and risk stratification in dilated cardiolaminopathies. J Am Coll Cardiol.

[B38] Andersen PS, Havndrup O, Hougs L, Sørensen KM, Jensen M, Larsen LA, Hedley P, Bie Thomsen AR, Moolman-Smook J, Christiansen M, Bundgaard H Diagnostic yield, interpretation, and clinical utility of mutation screening of sarcomere encoding genes in Danish hypertrophic cardiomyopathy patients and relatives. Hum Mutat.

[B39] Al-Mahdawi S, Chamberlain S, Cleland J, Nihoyannopoulos P, Gilligan D, French J, Choudhury L, Williamson R, Oakley C (1993). Identification of a mutation in the beta-cardiac myosin heavy chain gene in a family with hypertrophic cardiomyopathy. Br Heart J.

